# The effects of sugar in drinking water on *Streptococcus pyogenes* colonisation in a murine nasopharyngeal infection model

**DOI:** 10.1038/s41598-022-22648-5

**Published:** 2022-10-21

**Authors:** Farina Nor Hashimi, Julie Bennett, Michael G. Baker, Nicole J. Moreland, Troy L. Merry, Jacelyn M. S. Loh

**Affiliations:** 1 Department of Molecular Medicine and Pathology, School of Medical Sciences, University of Auckland, Private Bag 92019, Auckland, 1023 New Zealand; 2 Department of Nutrition, School of Medical Sciences, University of Auckland, Auckland, 1023 New Zealand; 3 Department of Public Health, University of Otago, Wellington, New Zealand; 4 Maurice Wilkins Centre for Molecular Biodiscovery, Auckland, New Zealand

**Keywords:** Bacteria, Risk factors, Bacterial infection

## Abstract

The number of sugar-sweetened beverages consumed per day has been associated with an increased risk of acute rheumatic fever, an autoimmune disease triggered by superficial *Streptococcus pyogenes* infection. To explore if there could be a biological basis for this association, we used a mouse model of *S. pyogenes* nasopharyngeal colonisation combined with a dietary intervention. We observed an increased bacterial load in the nasopharynx of mice receiving sucrose drinking water post-infection, suggesting that high sucrose intake promotes *S. pyogenes* growth and/or survival. This provides new insight into the potential biological basis behind the association seen in humans.

## Introduction

*Streptococcus pyogenes* is a Gram-positive bacterium and causative agent of both superficial and invasive infections. Most common infections such as pharyngitis (“strep throat”) and impetigo (“school sores”) arise in young children and are often self-limiting. However, in at-risk individuals, these superficial infections are strongly associated with the autoimmune sequelae, acute rheumatic fever (ARF)^1^. Repeated infection with *S. pyogenes* can trigger recurrent episodes of ARF that can cause permanent damage to the heart known as rheumatic heart disease (RHD)^2^.

Although ARF/RHD have largely disappeared from most high-income countries, the burden remains high in many low-income countries and within Indigenous communities in New Zealand (NZ) and Australia. In NZ, Māori and Pacific children have a rate of 80 cases per 100,000 population, which represent some of the highest rates in the world^3^. In 2015, there were an estimated 34 million people living with RHD, and globally RHD is responsible for around 320,000 deaths annually^4^.

Between 2013 and 2017, a case–control study to identify potentially modifiable risk factors for ARF was conducted in NZ. The Rheumatic Fever Risk Factors (RF RISK) study examined organism, host and environmental factors with closely matched controls enabling robust evaluation of a wide range of potential risk factors^5^. Alongside risk factors associated with household crowding and access to healthcare, the study reported elevated ARF associated with the number of sugar-sweetened beverages. Of ARF cases, 57.4% consumed one or more sugar-sweetened drinks per day compared with 37.2% of controls (adjusted OR 2.34 CI 1.50–3.66)^6^. The association between ARF and consumption of sugar-sweetened beverages remained highly significant in the multivariable analysis (adjusted OR 2.00; 1.13–3.54) and did not appear to be a marker for poor diet associated with poverty^6^. Other diet-related variables such as low fruit and vegetable consumption were not significantly associated with disease.

Current understanding of how increased sugar consumption increases ARF risk is lacking. To examine whether increased intake of high sugar beverages might directly impact *S. pyogenes* growth or survival in the nasopharynx, we used an established murine model of nasopharyngeal colonisation.

## Results

To investigate the effect of high sucrose consumption on *S. pyogenes* colonisation, a dietary intervention of 50% sucrose in drinking water was introduced 10 days prior to intranasal infection with an *emm*75 *S. pyogenes* strain. Mice in the control group (0% sucrose) were maintained on a standard diet prior to *S. pyogenes* infection. Respective diets were maintained in each group for a further 10 days-post infection during which bacteria burden was monitored by biophotonic imaging and culture.

Mice in the 50% sucrose group showed an average increase in water consumption of 6.2 ± 1.3 g per day (*p* = 0.0003, unpaired t-test) and an average decrease in food consumption of 1.7 ± 0.1 g per day (*p* < 0.0001, unpaired t-test) compared to controls which consumed an average of 3.6 ± 0.2 g water and 2.8 ± 0.1 g food per day. This equated to an average daily increase in energy intake of 14 ± 2.1 kcal (*p* < 0.0001, unpaired t-test) compared to control mice (8.6 ± 0.3 kcal). However, this increase in energy intake did not significantly alter the average increase in body weight of 0.7 ± 0.4 g (*p* = 0.0985, unpaired *t*-test) between the two groups over the study period (control increased from 21.43 to 22.48 g, 50% sucrose increased from 21.27 to 23.01 g). Average fasting blood glucose levels taken prior to infection (day-3) were also not significantly different (control = 6.5 ± 0.22 mmol/L, 50% sucrose = 6.25 ± 0.20 mmol/L; *p* = 0.3388, unpaired t-test).

**Figure 1:**
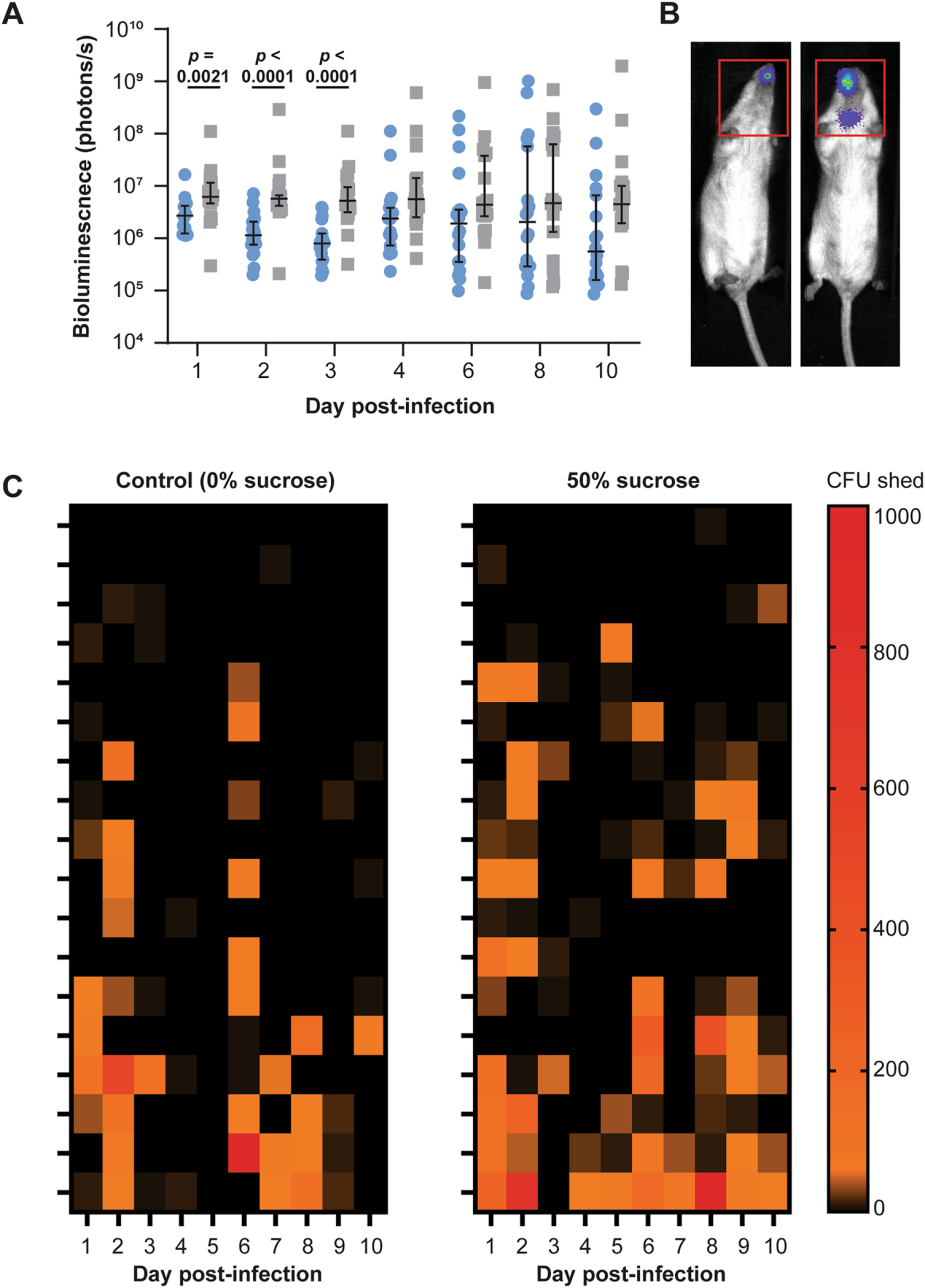
Sucrose in drinking water increases bacterial burden in the nasopharynx. FVB/n mice (n = 18 per group) were provided 0% (control) or 50% sucrose in drinking water ad libitum 10 days prior to nasal infection with *S. pyogenes*. (**A**) Bacterial burden was monitored by biophotonic live imaging on indicated days. Each data point represents an individual mouse in the control (blue circles) or 50% sucrose (grey squares) groups. Line indicates the median with 95% CI; *p* by Mann–Whitney test. (**B**) Representative image showing the region of interest included for the bioluminescent measurement used in A. (**C**) Bacteria shed from the nose was enumerated daily by standard culture techniques. Each row represents an individual mouse.

Bacterial burden measured by bioluminescence was significantly higher (*p* ≤ 0.0021, daily Mann–Whitney tests) in the treatment group compared to the control group for the first 3 days post-infection (Fig. 1A). This trend continued until the experimental endpoint, but was no longer significant at later timepoints (Fig. 1A). Bioluminescent signal was restricted to the nose of the control group, but was also detected in the throat of 2/18 mice in the 50% sucrose group (example in Fig. 1B). Nasal shedding of bacteria from the nose followed a similar trend to bioluminescent monitoring with more shedding observed in the 50% sucrose group (Fig. 1C). However, this trend was not significant.

## Discussion

We observed a significantly higher bacterial burden in the nasopharynx of mice given 50% sucrose in their drinking water compared to controls during the first 3 days post infection. This observation was mirrored at later timepoints but was no longer significant as the bacterial burden became more heterogenous in both groups. One limitation of using bioluminescence as a surrogate for bacterial burden is the high detection limit of 10^4^ CFU^7^. We therefore also used a second measure by assessing nasal shedding of bacteria by direct sampling. A corresponding, but non-significant trend was observed, where increased shedding was observed in the sucrose group.

How sucrose in drinking water increases the bacterial burden in the nasopharynx has not been addressed in this study, however, it may be that the presence of sucrose in the local environment provides optimal growth conditions for the bacteria. Indeed, *S. pyogenes* relies heavily on sugar fermentation for growth and energy production. Changes in environmental pH resulting from sugar fermentation can also lead to microcolony and biofilm formation, that are beneficial during the initial stages of infection^8^. Furthermore, sugar metabolism has also been linked to virulence gene regulation through directly affecting the activity of the Mga regulon^9^. The Mga regulon is a ubiquitous stand-alone regulator of *S. pyogenes* that influences approximately 10% of the *S. pyogenes* genome. These effects include regulation of important virulence genes such as *emm, fba, sclA,* and *scpA*, as well as those involved in sugar transport and utilisation^10^.

In our study, the primary bacterial burden was observed in the nose rather than throats of mice. While the nasal-associated lymphoid tissues (NALT) in the mouse are thought to be functionally equivalent to human tonsils^11^ and may act an important reservoir for bacteria, the location of the NALT in mice, on the posterior side of the palate^11^, begs to question the direct influence of sucrose at this location. As we noted an increase in energy intake in the sucrose group, we cannot exclude the possibility that our results are not a direct effect of sucrose, but rather a consequence of metabolic adaptions in these mice. This could be addressed in future studies by matching energy intake by pair feeding mice.

Interestingly, while previous research showed that consumption of sugar sweetened beverages was associated with increased risk of ARF^6^, consumption was not associated with increased risk of *S. pyogenes* skin and throat infection in humans^12^. Since our findings support the idea that sugary drinks promote *S. pyogenes* growth or survival in the nasopharynx, one could speculate that elevated bacterial burden maintained in a carrier state might lead to increased transmission within household members or increased chance of self-reinfection. This idea is supported by a recent classroom transmission study which pointed to ongoing transmission from asymptomatic carriers who are heavy shedders of bacteria^13^. Further research would be needed to investigate these potential pathways to disease.

## Methods

This study was performed in accordance with ARRIVE guidelines and approved by The University of Auckland Animal Ethics Committee ref. 08448. All methods were performed in accordance with the relevant guidelines and regulations.

Five- to eight-weeks-old male and female FVB/n mice (n = 18, bred in-house) were maintained in a temperature-controlled (20 °C) animal facility with 12 h light–dark cycle and ad libitum access to water and standard rodent chow (Teklad TB 2018; Harlan). Mice were assigned to control or sucrose groups by stratified randomization to balance age and gender between the two groups. Ten days prior to infection (day-10), drinking water of the sucrose group was replaced with 50% (500 g/L) sucrose water for the remainder of the experiment. Although standard sugar-sweetened beverages for human consumption typically contain ~ 10% sucrose, we chose a higher 50% sucrose concentration as a starting point based on expected nutritional outcomes previously described^14^. Mice were housed in sex-matched cages (2–3 per cage) with paper wool enrichment. Weight of water and food was measured every 2–3 days and the difference from previous measurement was used to calculate the average consumption per mouse. Blood glucose concentrations were measured using an Accu-chek performa on day-3 after a 6 h fast.

Animals were infected intranasally on day 0 with 10^8^ CFU *S. pyogenes* strain GAS13232 carrying the bioluminescent reporter plasmid pLZ12-Km2 P23R TA ffluc as previously described^15^. GAS13232 is a clinical isolate from a human throat swab, genotyped as *emm*75 (Institute of Environmental Science and Research, New Zealand). Bacterial burden was assessed by biophotonic imaging using an AMI HTX as previously described^16^. Aura software was used for image analysis. Direct nasal sampling was performed by gently pressing the mouse nose onto the surface of a brain heart infusion agar plate containing 200 μg/ml kanamycin ten times as previously described^16,17^. Samples were streaked out and incubated overnight at 37 °C for enumeration.

All statistical analysis was performed using GraphPad Prism software.

## Data Availability

The datasets generated during and/or analysed during the current study are available from the corresponding author on reasonable request.
